# Supplementary material for: Low light intensity delays vegetative phase change
Source: Plant Physiol. 2021 May 26;187(3):1177–88. doi: 10.1093/plphys/kiab243 (PMC8566249; doi:10.1093/plphys/kiab243)
Supplement: kiab243_Supplementary_Data [file kiab243_supplementary_data.pdf]

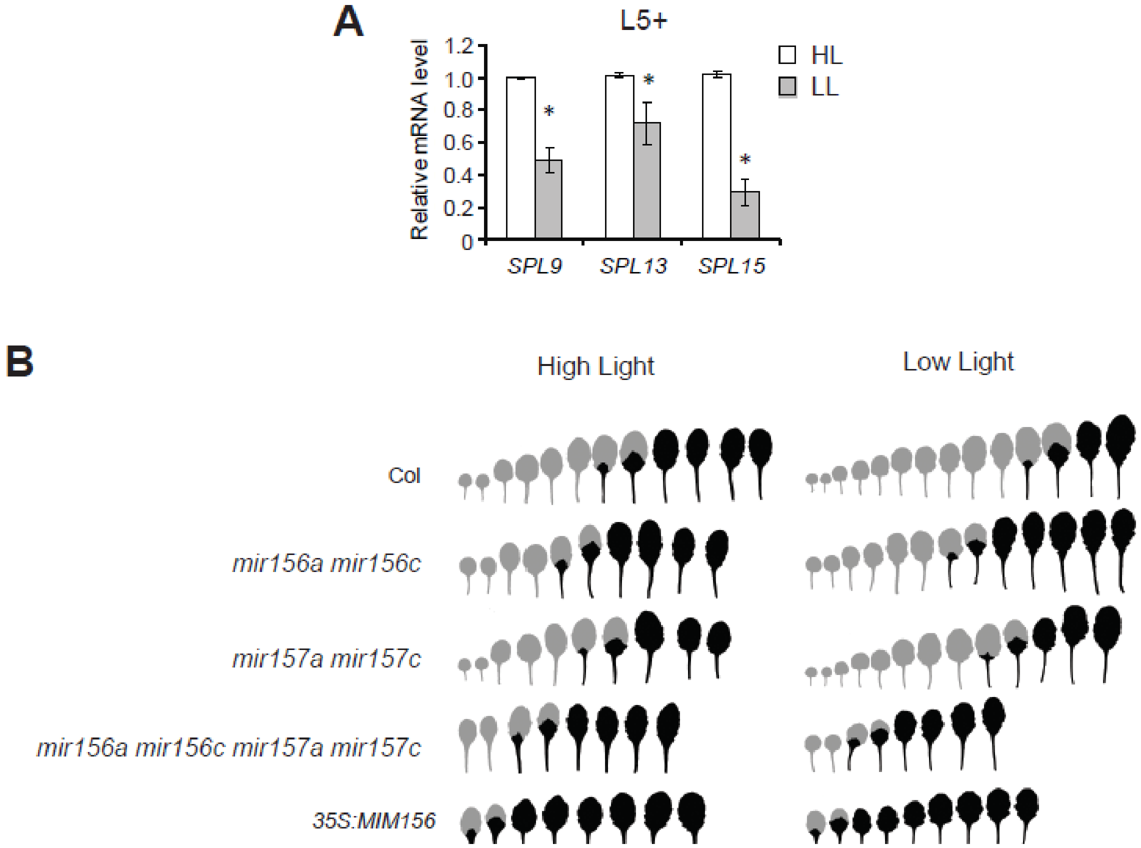

Supplemental Figure S1. LL delays vegetative phase change. (A) RT-qPCR analysis of SPL9, SPL13, and SPL15 transcripts in L5+ shoot apices from plants grown in HL and LL. *$p<0.05$, one-way ANOVA. Values are mean ± SEM from 3 biological replicates. (B) The morphology and pattern of abaxial trichome production in Col, *mir156a mir156c*, *mir157a mir157c*, *mir156a mir156c mir157a mir157c*, and *35S::MIM156* plants grown in HL and LL conditions. Grey = no abaxial trichomes. Black = abaxial trichomes. Images of leaves were digitally extracted for comparison.
